# Augmenting Instructional Animations with a Body Analogy to Help Children Learn about Physical Systems

**DOI:** 10.3389/fpsyg.2016.00860

**Published:** 2016-06-10

**Authors:** Wim T. J. L. Pouw, Tamara van Gog, Rolf A. Zwaan, Fred Paas

**Affiliations:** ^1^Department of Psychology, Education and Child Studies, Faculty of Social Sciences, Erasmus University RotterdamRotterdam, Netherlands; ^2^Department of Education, Utrecht UniversityUtrecht, Netherlands; ^3^Early Start Research Institute, University of Wollongong, WollongongNSW, Australia

**Keywords:** science education, body analogy, embodied learning, digital learning, gestures

## Abstract

We investigated whether augmenting instructional animations with a body analogy (BA) would improve 10- to 13-year-old children’s learning about class-1 levers. Children with a lower level of general math skill who learned with an instructional animation that provided a BA of the physical system, showed higher accuracy on a lever problem-solving reaction time task than children studying the instructional animation without this BA. Additionally, learning with a BA led to a higher speed–accuracy trade-off during the transfer task for children with a lower math skill, which provided additional evidence that especially this group is likely to be affected by learning with a BA. However, overall accuracy and solving speed on the transfer task was not affected by learning with or without this BA. These results suggest that providing children with a BA during animation study provides a stepping-stone for understanding mechanical principles of a physical system, which may prove useful for instructional designers. Yet, because the BA does not seem effective for all children, nor for all tasks, the degree of effectiveness of body analogies should be studied further. Future research, we conclude, should be more sensitive to the necessary degree of analogous mapping between the body and physical systems, and whether this mapping is effective for reasoning about more complex instantiations of such physical systems.

## Introduction

Instructional animations (IA) are increasingly implemented in educational environments (Chandler, 2009). The value of animated over static visualizations for instruction can be intuitively grasped: IA offer the learner *direc*t pick-up of process related information (i.e., information that interacts with time, such as causality and motion), which must be *inferred* from static visualizations (Spanjers et al., 2010). Surprisingly, empirical results concerning the effectiveness of IA are not as encouraging as these intuitions would predict. For example, in the instructional domain of physical systems (e.g., gears, electrical systems, etc.), although visual presentation benefits learning overall (as opposed to non-graphical instructions), findings regarding the effectiveness of animated versus static visualizations are mixed (Hegarty et al., 2003).

Based on the mixed results Tversky et al. (2002) concluded: “The many failures to find benefits of animation … calls for deeper inquiry into information processing of animation” (p. 255). This was taken to heart, and later studies have suggested that the main problem with learning from dynamic visualizations is that it imposes a high cognitive load on working memory from the learner due to information transience inherent to dynamically changing visualizations (Ayres and Paas, 2007a,b). To be effective, it is argued, the negative effects of transience in IA need to be counteracted, for instance, by means of cueing, or segmentation (Spanjers et al., 2010).

There is one type of task, however, for which IA consistently seem beneficial for learning compared to static visualizations even without measures to counteract transience. Namely, a meta-analysis (Höﬄer and Leutner, 2007) showed a small effect size of learning gains in animated vs. static visualizations under the condition that the instructional content involves learning bodily routines (e.g., origami, assembly, knot tying). It has been suggested that because human movement is automatically and efficiently processed by the cognitive system (we will return to this in the next section), the transience inherent in IA depicting such tasks may be counteracted (Van Gog et al., 2009).

Indeed, evidence is accumulating that the human cognitive system is distinctively attuned to the body, the body of others, and its possibilities for interactions (e.g., Rizzolatti et al., 1996; Amorim et al., 2006). For example, neuropsychological evidence suggests that perceived human body parts are distinctively processed in particular areas of the brain (extrastriate body area; Peelen and Downing, 2007) as compared to perceived body parts of non-human animals (Peelen and Downing, 2007). Moreover, human bodies are readily mapped onto one’s own body schema (Semenza and Goodglass, 1985; Van Gog et al., 2009). For instance, mental rotation of shapes represented as a body is performed faster than mental rotation of inanimate objects (Amorim et al., 2006).

Therefore, in the present paper we investigate whether augmenting IA with a body analogy (BA) improves learning about non-human movement content (originally proposed by De Koning and Tabbers, 2011). Specifically, we investigate whether the effectiveness of IA might be improved by augmenting the learning content (in this study: class 1 lever problems) with a BA. We hypothesize: by meaningfully mapping a physical body on a physical system during instruction, a less cognitively demanding route of knowledge-transfer might be created (as opposed to learning about inanimate objects). “Less demanding,” as learners readily map bodily actions on their own body schema. Moreover, learners are very familiar with forces acting on the body, which can be used as an analogy for forces acting on physical systems.

There is evidence already that the body can be mapped on physical systems. For example, when children or adults convey their knowledge about a particular topic they often use gestures that are meaningfully related to the topic’s content (e.g., Goldin-Meadow and Momeni-Sandhofer, 1999; Garber and Goldin-Meadow, 2002; Goldin-Meadow and Alibali, 2002; Hutchins and Nomura, 2011). Importantly, gestures do not simply mirror what is expressed in speech. Rather, gestures can accommodate and complement what is expressed verbally with idiosyncratic information expressed in gesture alone. For instance, in a study by Pine et al. (2004) co-speech gestures that emerged when children explained the workings of a class 1 lever (balance beam) were analyzed (see also Pine and Messer, 2000). To solve lever (e.g., a balance-beam) problems children must attain knowledge about the effects of (I) weights, (II) distance of the weight from the fulcrum, and (III) the positioning of the fulcrum. About one-third of the children (5–9 years) explaining the solution to a balance-beam problem produced gesture-speech mismatches. Children verbally explained the solution to the problem in terms of one property (e.g., I; talking about the weights on the beam), while concurrently expressing another (more advanced property) in gesture (e.g., III; expressing the position of the fulcrum in gesture). Even more remarkably, those children that produced mismatches as compared to those that did not, were more likely to improve on pre- to post-test measures of learning. If knowledge about physical systems develops in sensori-motor modalities as research on gesture suggests, augmenting the learning content with sensori-motor stimuli might improve learning (Höﬄer and Leutner, 2007; Van Gog et al., 2009; Pouw et al., 2014).

Yet, it seems to be the case that augmenting IA about physical systems with sensori-motor information may be suitable for some but not for others (Zacharia et al., 2012; for an overview, see Pouw et al., 2014). For example, kindergartners’ learning about balance beams improved when they were given opportunities to physically interact with a balance beam (class 1 lever), but only when they possessed an incorrect preconception of how a balance beam works (Zacharia et al., 2012). This suggests that especially those with incomplete understanding of a physical system are aided by additional body-analogous information. Therefore, it is important to take into account learners cognitive predispositions when investigating the instructional potency.

### Present Study

**FIGURE 1:**
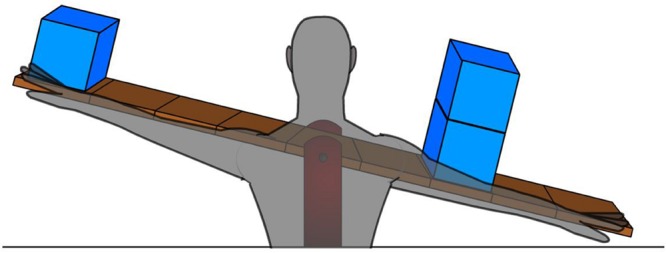
**A snapshot of the instructional animation in the BA condition (the seesaw will balance out in this example)**.

In the present study, primary school children learned from IA about a class 1 lever (a seesaw). The workings of levers can be considered as a classic context to test children’s conceptual and procedural learning processes about physical systems (Karmiloff-Smith and Inhelder, 1974; Dixon and Dohn, 2003; Pine et al., 2004). We designed an IA (duration 6.5 min) in which relevant concepts for understanding the working of a seesaw were demonstrated, such as weights, balance, fulcrum, and mechanical advantage. Half of the sample was confronted with a ‘BA IA’ in which a transparent body was projected onto the seesaw (**Figure 1**: BA condition) and the other half were given the same IA without this BA (control condition). The body provided an analogy of the concept of mechanical advantage: objects placed further from the fulcrum (analogy: joint) will exert more force than objects placed closer to the fulcrum. Furthermore, if similar weights are put at similar places on the arm they will feel equally heavy (balance) or when they are located at different places, they will not feel equally heavy (disbalance).

Learning performance was assessed through a three choice reaction-time task that assessed accuracy and speed of determining whether a seesaw will pivot to the left or the right, or will balance out, given different configurations of the weights, and the positions of the weight relative to the fulcrum. Additionally, we confronted children with a similar three-choice transfer task that consisted of new concepts, such as interconnecting seesaws, or replacement of the fulcrum.

We hypothesized that the BA condition as compared to the control condition would show better learning overall (i.e., higher accuracy, faster solving speed on the test tasks). Importantly, to minimize individual cognitive differences between conditions we semi-randomly assigned conditions based on general math scores of the children. We used children’s math scores as they are closely related to learning about physical systems, and have been found to strongly correlate with their visuospatial working memory capacity (e.g., Van der Ven et al., 2013), which directly relates to issues of cognitive load associated with IA (Ayres and Paas, 2007a,b). Per exploration we also investigate whether general math skill interacted with the effectiveness of the conditions, as it might be an important cognitive predisposition for learning in the current domain. We also measured subjective experiences of cognitive load, by asking children to rate how much mental effort they invested and how difficult they found the tasks. In addition, we asked them to rate how interesting they found the tasks, which could give an indication of differences in cognitive engagement.

## Materials and Methods

### Participants and Design

This study was conducted in accordance with the guidelines of the ethical committee of the institute of psychology at Erasmus University Rotterdam. All children participated based on parental informed consent, where information about the study was provided 2 weeks prior to the experiment and parents were given the opportunity to withdraw their child from participating. A total of 74 Dutch primary school children (three classrooms from two separate schools) were tested (mean age, 12.49, *SD* = 0.54; range 10–13; 51.4% female). The two IA-conditions were: control (*N* = 36, 52.8% female) vs. BA (*N* = 38, 50% female). Children were pseudo randomly assigned (see **Table 1** for frequencies) to condition by matching for level of general math skill as measured by the national standardized Cito math test or (in one school) an equivalent standardized test that assigns the children to comparable levels of skill as the Cito test does. From highest to lowest, these are: A (highest 25%), B (next 25%), C (next 25%), D (next 15%), and E (lowest 10%). This test was taken within the school-semester year in which the experiment took place, and the children’s scores were provided by the schools.

**Table 1:** Number of participants per condition and general math skill.

	Control condition	BA condition
A	7	8
B	12	12
C	7	8
D	8	7
E	2	3
Total	36	38

### Materials

#### Instructional Animations

The IA^1^ were designed in Adobe Flash Professional CS 5.5. The voice-over and textual instructions were programmed in ActionScript 3.0 (IA’s^2^). The IA consisted of an introduction to the basic concepts of class 1 levers narrated by a female voiceover and explained with a dynamic visualization of a seesaw. In the first part of the IA (3.5 min), basic concepts such as the fulcrum, left and right arm of the seesaw, (dis)balance, weights, and mechanical advantage was introduced. Throughout the instruction no explicit information was provided about formulas related to the constructs. For example, mechanical advantage was explained by showing a balanced seesaw in a mechanical advantage state, with the voiceover instruction informing learners that: “The heavy weight is twice as heavy as the lighter weight, but the seesaw is still in balance! This is because the distance of the heavy weight is two times closer to the fulcrum than the lighter weight” (for further instructions^2^). The second part of the IA was not narrated and consisted of 24 trials (3 min) that showed different configurations of weights on varying positions from the fulcrum and its effect on the seesaw (tilt left, right, or balance).

For the BA condition the only difference in the IA as compared to the control condition was that a transparent human body was additionally projected over the seesaw (i.e., no differences in narrated instruction). Importantly, the arms of the projected body moved together with the movement of the seesaw (**Figure 1**). Only once in the narration (but in both conditions) a reference was made to how it would feel to have weights on one’s actual arms. This reference was made after the explanation of mechanical advantage, which showed a seesaw balancing out with unequal amount of weights (**Figure 1**). This was done to ensure that children in the BA condition would be more likely to see the relevance of the body projected over the seesaw.

#### Reaction-Time Task

**FIGURE 2:**
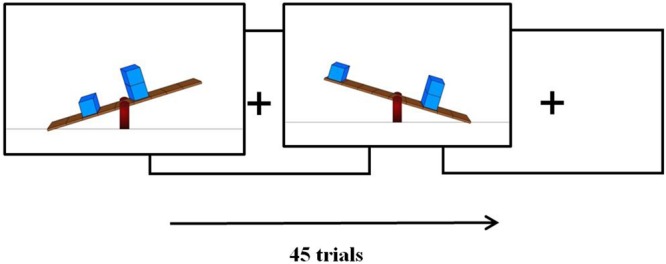
**Example of two reaction time trials.** Note that trials were given the initial state of the seesaw randomly and the children answered with button presses what the correct state of the seesaw would be (pivot left, balance, pivot right).

A three choice reaction-time task was developed (programmed in E-prime) to assess children’s accuracy (number of correct responses) and speed (reaction-time) in solving class-1 lever problems. The RT task consisted of 45 trials (and three practice trials) in which children had to judge whether a seesaw would balance, or tilt down to the left or to the right. Each trial showed a seesaw with one or two blocks on either side of the arms of the seesaw on deferring distances from the fulcrum (**Figure 2**). The number and location of the weight varied for these 45 trials. Children were required to determine which way the seesaw would tilt, or whether it would attain balance, regardless of the current state of the seesaw (i.e., tilted to left/right or balanced). We varied the initial state of the seesaw randomly as to prevent any spurious effects of the initial state of the seesaw on accuracy and speed. Children responded by pressing on a QWERTY keyboard, “P” if the seesaw would tilt to the right, “Q” if it would tilt to the left and SPACE if the seesaw would be in balance.

#### Transfer Task

**FIGURE 3:**
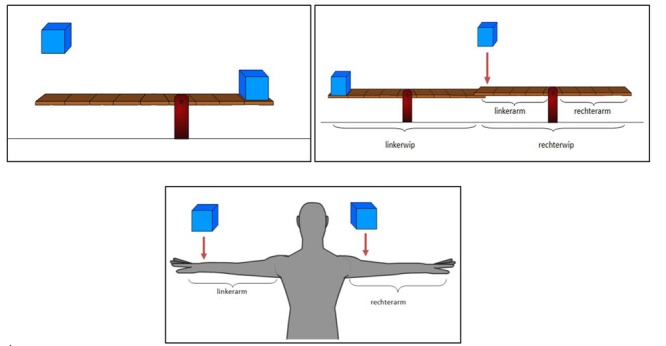
**Example of three transfer task problems.** In the first example (left above), children were asked to “Judge whether the seesaw pivots to the left, remains in balance, or pivots to the right.” This was the same for the second example (right above), but then for the right seesaw [rechterwip]. In the third example children were asked to “which weight will feel the heaviest for this person, or will the weights feel just as heavy?”. Note that the terms left arm [linkerarm] and right arm [rechterarm] were added for reference, as learned during the IA.

The transfer task, consisting of 15 lever-problems, aimed to assess children’s ability and solving speed to further apply the principle of mechanical advantage on new or more complex problems. Twelve problems required children to judge what the end-state would be (tilt left, right, or balance) of a particular seesaw in a set of two interconnected seesaws, in four of those trials the fulcrum was not placed in the center (**Figure 3**). The last three problems required children to predict how these forces would act on the body (e.g., how heavy a block would feel when placed on the arms, or which seesaw needed to be pushed down the hardest given a number of weights on the seesaws).

#### Mental Effort, Difficulty, Interest

We obtained ratings of experienced mental effort, interest, and perceived difficulty of the IA, RT-task, and the transfer task directly after completion. Children answered on a 5-point scale “How hard did you need to think to understand the previous video/task” (mental effort; 1 = ‘not hard,’ to 5 = ‘very hard’), “How interesting did you find this previous video/task” (interest; 1 = ‘not interesting,’ to 5 = ‘very interesting’) and “How difficult did you find this previous video/task” (difficulty; 1 = ‘not difficult,’ to 5 = ‘highly difficult’).

#### Demographics

Information on age, sex, and Cito test score of general math skill of the children were provided by the schools.

### Procedure

Children were tested one or two at a time, in a quiet room at their school. If children were tested at the same time the two experimenters ensured that children did not face each other directly and that there was enough distance between them so that they were not disturbed in any way. Children were seated in front of a laptop and were informed that they would watch an instructional video and perform two tasks to assess what they had learned. They were subsequently asked to put on the headphones so that the experimenter could start the video. Subsequently, children performed the reaction-time task and were instructed to do so “as fast and accurate as possible.” Beforehand, children were given three easy practice trials which the experimenter could repeat if needed to ensure they understood the task. Subsequently, children were confronted with the transfer task that was provided in a booklet and they could solve at their own pace (i.e., speed was not emphasized as in the RT-test task). The experimenter used a stopwatch to assess overall solving speed. Immediately after watching the IA, performing the RT, and solving the transfer task, children completed the subjective ratings of effort, interest and difficulty that were printed on a sheet of A4 paper per task. All children received a small present for their participation (handed out in class on the last day of testing).

### Data Analyses

Accuracy and RT-scores for the transfer task and RT-task more than 2 SD from the overall-mean were treated as outliers and were excluded from the analysis (reported in the Results section when applicable).

#### Reaction-Time Task

Performance accuracy was measured by summing the correct answers on 45 trials (range: 0–45) and speed was measured by computing the mean reaction time (in ms) on correct trials.

#### Transfer Task

Performance was measured by summing the correct answers on 15 trials (range: 0–15) as well as overall solving speed in seconds.

## Results

### Mental Effort, Difficulty, Interest

Data are presented in **Table 2**. *T*-tests showed no significant differences between conditions in self-reported mental effort, difficulty, or interest, on the IA, RT-task, or transfer task.

**Table 2:** Means and SDs per condition and task-phase for mental effort, interest, and difficulty.

Condition		Instructional animation		Reaction-time task		Transfer task	
		*M*	*SD*	*M*	*SD*	*M*	*SD*
Control condition	Mental effort	1.81	1.064	2.25	1.05	2.50	1.00
	Interest	3.56	1.319	3.58	1.36	3.69	1.33
	Difficulty	1.92	1.13	2.58	1.16	2.64	1.10
BA condition	Mental effort	1.79	0.935	2.26	1.155	2.27	1.03
	Interest	3.45	1.350	3.74	1.178	3.26	1.35
	Difficulty	2.11	1.23	2.37	1.08	2.71	1.04

### RT-Task Performance

#### Accuracy

The overall accuracy score on the RT-task was 59.91% (*M* = 26.96, *SD* = 5.56). Four participants scored < 2 SDs below the mean (i.e., <15) and were therefore excluded from the analyses (no participants scored > 2 SD). This resulted in an analysis on data of 70 participants, with *N* = 34 in the control condition (*N* = 7 on math skill level A, *N* = 11 on level B, *N* = 7 on level C, *N* = 8 on level D, and *N* = 1 on level E), and *N* = 36 in the BA condition (*N* = 8 scoring on math skill level A, *N* = 12 on level B, *N* = 8 on level C, *N* = 6 on level D, and *N* = 2 on level E).

We performed a multiple stepwise regression to assess main effects of math skill and condition and its potential interaction. First, we entered math skill (recoded for analysis, E = -2, D = -1, C = 0, B = 1, and A = 2; higher scores means higher math skill) which was a significant predictor, *F*(1,68) = 17.256, *p* < 0.001, explaining 19.1% of the variance (based on *R*^2^_adjusted_), with higher math skill resulting in higher accuracy, β = 0.450, *t*(68) = 4.154, *p* < 0.001. The effect of condition was assessed by adding condition as a predictor for RT accuracy into a stepwise regression after math skill. Condition was coded as 0 for the control condition and 1 for the BA condition. The overall model remained significant, *F*(2,67) = 9.417, *p* < 0.001, explaining 19.6% of the variance in RT-accuracy. Condition was a positive but non-significant predictor for RT-accuracy, β = 0.130, *t*(67) = 1.208, *p* = 0.231, *R*_Partial_ = 0.146. Math skill remained a significant predictor, β = 0.429, *t*(68) = 4.136, *p* < 0.001, *R*_Partial_ = 0.451.

**FIGURE 4:**
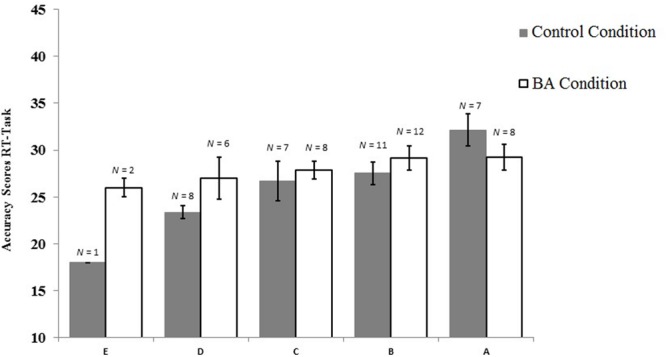
**Accuracy scores and standard error per condition and general math skills (E = lower and A = higher general math skill)**.

We further assessed whether general math skill moderated the effect of condition by adding an interaction term of condition and math skill into the regression model. This resulted in significant model-fit, *F*(3,66) = 8.533, *p* < 0.001, explaining 24.7% of the variance in RT accuracy. General math skill remained a significant predictor, β = 0.704, *t*(66) = 4.645, *p* < 0.001, *R*_Partial_ = 0.496, and now condition was significantly positively related with RT accuracy, β = 0.230, *t*(66) = 2.040, *p* = 0.045, *R*_Partial_ = 0.244. Furthermore, there was a significant interaction, β = -0.371, *t*(66) = 2.346, *p* = 0.022, *R*_Partial_ = -0.277, indicating that children with lower math skill were more likely to be positively affected by the BA condition (in terms of RT-accuracy) than those with higher math skill (**Figure 4**).

#### Speed

**FIGURE 5:**
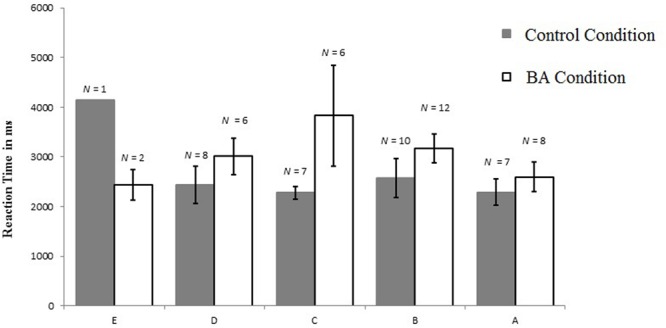
**Mean reaction times and standard error for the RT-task per condition and general math skills (E lowest score, A highest score on general math skill)**.

The overall mean reaction time on correct trials was 2791 ms (*SD* = 1331). Three additional participants were excluded from the analyses as their data fell over 2 SDs above the mean (>5453 ms; no participants scored < 2 SD). This resulted in an analysis (also see **Figure 5**) on data of 67 participants, with *N* = 33 in the control condition (*N* = 7 scoring on math skill level A, *N* = 10 on level B, *N* = 7 on level C, *N* = 8 on level D, and *N* = 1 on level E), and *N* = 34 in the BA condition (*N* = 8 scoring on math skill level A, *N* = 12 on level B, *N* = 6 on level C, *N* = 6 on level D, and *N* = 2 on level E). Math skill (recoded E = -2, D = -1, C = 0, B = 1, and A = 2) was not a significant predictor, *F*(1,65) = 0.327, *p* = 0.569, *R*^2^_adjusted_= -0.010, showing a non-significant relation with speed on correct RT trials β = -0.071, *t*(65) = -0.527, *p* = 0.569. We added condition together with general math skill as a predictor for speed on correct trials into the stepwise regression model. The overall model-fit was non-significant, *F*(2,64) = 2.878, *p* = 0.064, *R*^2^_adjusted_= 0.054, math skill remained a non-significant predictor, β = -0.083, *t*(64) = -0.695, *p* = 0.490, *R*_Partial_= -0.083, and condition was a positive significant predictor, with children in the BA condition being slower on correct trials overall, β = 0.279, *t*(64) = 2.325 *p* = 0.023, *R*_Partial_ = 0.279. To assess a possible interaction effect we entered the interaction term of condition and math skill into the regression model, this yielded no significant results, nor a greater fit of the model.

#### Speed–Accuracy Trade-off

Additionally, for exploratory purposes we assessed whether there was a speed–accuracy trade-off by calculating an inverse efficiency measure (IES; Townsend and Ashby, 1978; e.g., Setti et al., 2009), IES = 
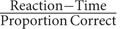
. A higher score entails a more extreme association of speed and accuracy, where slower reaction times are associated with a higher proportion of correct responses or faster reaction time with a higher proportion of incorrect responses.

The overall mean IES was 5709 ms (*SD* = 2870). Four participants were excluded from this analysis as their scores fell 2 SDs above the mean (>11450 ms; no participants scored < 2 *SD*). The resulting sample consists of 70 participants, with *N* = 34 in the control condition (*N* = 7 scoring on math skill level A, *N* = 12 on level B, *N* = 7 on level C, *N* = 8 on level D, and *N* = 0 on level E), and *N* = 36 in the BA condition (*N* = 8 scoring on math skill level A, *N* = 11 on level B, *N* = 7 on level C, *N* = 7 on level D, and *N* = 3 on level E).

Math skill (recoded E = -2, D = -1, C = 0, B = 1, and A = 2) did not predict IES, *F*(1,68) = 1.814, *R*^2^_adjusted_= 0.013, β = -0.161, *t*(68) = -1.347, *p* = 0.183. Adding condition next to math skill as a predictor of IES resulted in a non-significant overall model-fit, *F*(2,67) = 1.445, *p* = 0.243, *R*^2^_adjusted_= 0.012; math skill remained non-significant, β = 0.124, *t*(67) = 1.037, *p* = 0.304, *R*_partial_ = -0.155, and condition was also a non-significant predictor of IES, β = -0.154, *t*(64) = -1.283 *p* = 0.204, *R*_Partial_ = 0.126. Adding an interaction term of the previous set of predictors (math skill and condition) did not yield a significant fit of the model, *F*(3,66) = 1.258, *p* = 0.296, *R*^2^_adjusted_= 0.011; the interaction term was a non-significant predictor, β = 0.181, *t*(66) = 0.942 *p* = 0.350, *R*_Partial_ = 0.115, and adding it did not affect results regarding math skill, β = -0.291, *t*(66) = -1.542 *p* = 0.128, *R*_Partial_ = -0.186, or condition, β = 0.078, *t*(66) = 0.599 *p* = 0.551, *R*_Partial_ = 0.074, in relation to IES.

### Transfer Task Performance

#### Accuracy

The overall accuracy on the transfer task was 49.62% (*M* = 7.38, *SD* = 1.90. Two participants performed < 2 SDs below the mean (<3.58; no participants scored > 2 SD) and were therefore excluded from the analyses. This resulted in an analysis on data of 72 participants, with *N* = 36 in the control condition (*N* = 7 scoring on math skill level A, *N* = 12 on level B, *N* = 7 on level C, *N* = 8 on level D, and *N* = 2 on level E), and *N* = 36 in the BA condition (*N* = 8 scoring on math skill level A, *N* = 12 on level B, *N* = 8 on level C, *N* = 6 on level D, and *N* = 2 on level E).

A regression analysis showed that math skill (recoded E = -2, D = -1, C = 0, B = 1, and A = 2) was a significant predictor of transfer task performance, *F*(1,70) = 7.320, *p* = 0.009, *R*^2^_adjusted_ = 0.082, showing a positive relation with performance β = 0.308, *t*(70) = -0.2.706, *p* = 0.009.

We added condition after math skill as a predictor for transfer task performance into the stepwise regression model. The overall model-fit remained significant, *F*(1,69) = 3.697, *p* < 0.05, *R*^2^_adjusted_ = 0.071. Math skill remained a significant predictor, β = 0.310, *t*(69) = 2.705, *p* < 0.01, *R*_Partial_ = 0.310. Condition was not a significant predictor, β = -0.403, *t*(68) = -0.403, *p* = 0.688, *R*_Partial_ = -0.048. We further added an interaction term of condition and general math skills into the stepwise regression model, but this resulted in a model with only non-significant predictors (*p* > 0.246).

#### Speed

The overall mean solution speed on the transfer task was 308 s (*SD* = 77.11). Two additional participants were excluded from the analyses as their data fell 2 SD’s from the mean (>462 s; no participants scored < 2 SD). This resulted in an analysis on data of 70 participants, with *N* = 34 in the control condition (*N* = 6 scoring on math skill level A, *N* = 11 on level B, *N* = 7 on level C, *N* = 8 on level D, and *N* = 2 on level E), and *N* = 36 in the BA condition (*N* = 8 scoring on math skill level A, *N* = 12 on level B, *N* = 8 on level C, *N* = 6 on level D, and *N* = 2 on level E). We first assessed whether math skill predicted overall speed on Transfer task in a regression analysis. Math skill was not a significant predictor, *F*(1,68) = 0.520, *p* = 0.520, *R*^2^_adjusted_= -0.007, β = -0.087, *t*(68) = -0.72, *p* = 0.473. We added condition next to general math skill as a predictor for speed on transfer task into the stepwise regression model. The overall model-fit was not significant, *F*(2,67) = 1.699, *p* = 0.395, *R*^2^_adjusted_= 0.020. Math skill remained a non-significant predictor, β = -0.102, *t*(67) = -0.856, *p* = 0.395, *R*_Partial_ = -0.104, and condition was a non-significant predictor on solving speed on the transfer task, β = 0.202, *t*(67) = 1.692, *p* = 0.095, *R*_Partial_ = 0.202. We obtained no significant results when entering an interaction term after math skill and condition.

#### Speed–Accuracy Trade-off

For exploratory purposes we assessed whether a speed–accuracy trade-off could be detected using the inverse efficiency measure (IES; Townsend and Ashby, 1978), IES = 
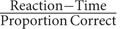
. As a reminder, a higher score entails a more extreme association of speed and accuracy, where slower reaction times are associated with a higher proportion of correct responses, or faster reaction times with a higher proportion of incorrect responses. Overall mean IES for the transfer task was 674.41 s (*SD* = 256.94). Three participants’ scores fell above 2 SDs above the mean (>1188; no scores < 2 SD), and were excluded. The resulting sample contained scores of 71 participants, with *N* = 34 in the control condition (*N* = 7 scoring on math skill level A, *N* = 12 on level B, *N* = 6 on level C, *N* = 7 on level D, and *N* = 2 on level E), *N* = 37 (*N* = 8 scoring on math skill level A, *N* = 12 on level B, *N* = 8 on level C, *N* = 7 on level D, and *N* = 2 on level E).

**FIGURE 6:**
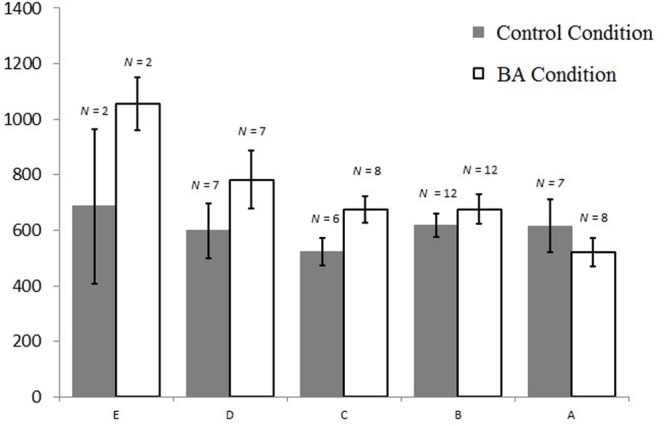
**Mean inverse efficiency scores and standard errors for the transfer task per condition and general math skills (E lowest score, A highest score on general math skill)**.

Interestingly, math skill was predictive for IES, model fit *F*(1,69) = 5.334, *R*^2^_adjusted_ = 0.058, β = -0.268, *t*(69) = -2.310, *p* = 0.183, with lower math skill being associated with a lower inverse efficiency score. Adding condition as a predictor next to math skill slightly improved the overall model fit, *F*(2,68) = 4.234, *p* = 0.018, *R*^2^_adjusted_ = 0.085, with math skill remaining a significant predictor of IES, β = -0.269, *t*(68) = -2.356 *p* = 0.021, *R*_Partial_ = -0.275, but condition was a non-significant predictor of IES, β = 0.179, *t*(68) = 1.726 *p* = 0.089, *R*_Partial_ = 0.205. Subsequently we added the interaction term next to math skill and condition, which substantially improved the overall model fit, *F*(3,67) = 5.077, *p* = 0.003, *R*^2^_adjusted_ = 0.149. In this more predictive moderation model, math skill no longer had a significant main effect on IES, β = 0.011, *t*(67) = 0.071, *p* = 0.943, *R*_Partial_ = 0.009, but condition had a significant main effect on IES, β = 0.301, *t*(67) = 2.552, *p* = 0.013, *R*_Partial_ = 0.298, which was further qualified by a significant interaction effect, β = -0.406, *t*(67) = -2.457, *p* = 0.016, *R*_Partial_ = -0.289 (**Figure 6**). These results support a moderating effect of condition and math skill, such that children in the BA condition were more likely to make less mistakes with slower solving speeds (or more mistakes with faster speeds), especially for those children with a lower math skill score.

## Discussion

We investigated whether children’s learning benefited from augmenting an IA about class 1 levers with a BA. It was found that when taking general math skill into account as a moderator, this BA condition was positively affecting lever problem-solving accuracy on the RT-test as compared to the control condition, in which the same instructional animation was shown without the BA. However, this effect was qualified by an interaction, showing that the BA condition improved accuracy on the RT-test for children with a lower level of general math skill, and was absent (if not reversed) for children with a higher math skill. Finally, no evidence was obtained for performance benefits on the transfer task.

As the results are mixed, the question arises whether the BA was “analogous enough” to be informative for learning. Indeed, there are important differences between a seesaw and the BA. Most notably, the BA is imperfect, as the body has two joints with independent moving arms whereas the seesaw has one fulcrum with movement of the arms that are co-dependent. Such (and possibly other) differences might interfere with properly understanding mechanics of seesaws. However, there is some information in the BA that directly corresponds with the mechanics of the seesaw. Namely, there is a one to one correspondence to the difference in weight that would be felt when placing blocks on one’s arm with that of the direction of pivot of the seesaw. For example, placing one block on the left arm of the seesaw near the fulcrum and one block on the right arm away from the fulcrum will result in a pivot to the right due to mechanical advantage. This directly corresponds with the relative difference in weight that would be felt when placing one block on the left arm near the fulcrum (joint) and one block on the right arm away from the fulcrum (also due to mechanical advantage). Indeed, in the voiceover of the instructional animations we emphasized to the learner that this was a relevant correspondence.

Therefore, we speculate that the body-analogous information that was present provided a possible means to process the learning content by activating implicit motor knowledge, which provided those children that are least receptive to learning about abstract content (i.e., those with a lower general math skill) a way to ground unfamiliar force-dynamics of the seesaw in familiar force-dynamics of the body. In line with observations made by Van Gog et al. (2009), this grounding would be established through mapping of the model’s body onto one’s own body. Indeed, it seems that when a rule or process is already understood, additional grounding in concrete experiences is unnecessary (Zacharia et al., 2012). We further speculate that in the current case children benefited from the BA condition when performing the reaction time task because they were mentally *simulating* the force dynamics related to the body (see Van Gog et al., 2009), i.e., mentally re-enacting the learned correspondences of the body and the lever for more accurate problem solving. Yet, we did not find a similar effect of condition on transfer task accuracy as we did in on the reaction time-task. This signals that an efficient strategy on one task does not always readily transfer to another. As shown by Dixon and Dohn (2003), when solving problems with interconnecting balance beams (also included in our transfer task), problem solvers may use more abstract strategies (i.e., alternating strategy; see Dixon and Dohn, 2003 for details) than simply judging each state of each seesaw to judge its effect on the next connected seesaw. Perhaps, judging the forces of a single seesaw like in the RT-task is aided by a BA, whereas this strategy might prove inefficient for solving the transfer problem. On the interconnected seesaw problem, more abstract strategies are more efficient, and discovery of these abstract strategies might even be hampered by a strategy based on a BA. Some evidence speaks to this interpretation as we found that children with lower math scores in the BA condition (as opposed to the control condition) showed higher speed–accuracy trade-offs, indicating that when these children responded faster their performance was lower (or was higher when they responded more slowly). This might indicate that they attempted to use the BA, but that this was a time-consuming and therefore not necessarily more efficient strategy for solving the transfer task, especially for children with a lower math skill (who would be more likely to use the BA). In sum, future research should be sensitive to the kind of strategy a particular BA solicits, and on which tasks that strategy could be expected to help learning.

Furthermore, in line with findings on the expertise reversal effect (Kalyuga, 2007), the accuracy results show that this mental simulation on the reaction time test was only helpful for children with lower math ability (lower visuospatial working memory capacity) but not helpful, or potentially even detrimental, to those with higher ability (working memory capacity). Perhaps those with a higher ability did not require additional help to induce rules from physical systems so that for them mental simulation during the test task evoked by the BA is superfluous and possibly distracting process. Perhaps this explains why no effects on the transfer test were found, as it was more difficult to use one’s own body as an analogy on most of the test items that involved multiple balance beams.

It should be noted that the present study provides more of a *demonstration* than an *elaboration* of how a BA can affect learning. Indeed, the current design has some shortcomings that prevent such elaboration. For instance, although this task was not one taught in school, the possibility cannot be excluded that some children had more prior knowledge than others; and our current design did not allow for assessing learning gains, as we did not provide children with a pre-test. Furthermore, the current results do not allow us to determine whether higher learning outcomes of children with lower math scores in the BA condition were indeed achieved because cognitive load related to transience was counteracted by more efficient processing due to the BA. There were no differences in mental effort or difficulty ratings between the conditions, but this does not necessarily mean that cognitive load imposed by transience was not reduced. Perhaps the cognitive capacity that was freed-up by reducing the load imposed by transience, was used for processes that were effective for learning, thereby resulting in an similar experience in cognitive load. Future studies might investigate the underlying cognitive mechanisms in more detail, for instance by using continuous and objective cognitive load measures that can be connected to events in the animation such as dual task measures that do not interfere with animation processing (e.g., Park and Brünken, 2014) or EEG measures (e.g., Antonenko et al., 2010).

Future research should further focus on (a) the potential difference in receptivity of children with different individual cognitive capacities for learning with body analogies, (b) the scope of the effectiveness of body analogies on other physical systems (e.g., electrical circuits, gear systems, see for example Johnson-Glenberg et al., 2015), or even more abstract learning domains such as grammatical or language learning (e.g., Lu, 2011), and (c) finally the precise cognitive processes underlying this type of learning (e.g., Brucker et al., 2015).

## Conclusion

Despite some limitations, our finding that a relatively simple modification of the instructional animation via a BA imbued a positive effect on performance, especially for those with lower general math skill, is a very promising result for future applications in educational practice.

## Data Access

Data supporting this research report can be retrieved from the Open Science Framework^3^.

## Author Contributions

WP designed and collected the data; TvG, RZ, and FP co-designed the experiment. WP written, and TvG, RZ, and FP provided critical revisions on the manuscript.

## Conflict of Interest Statement

The authors declare that the research was conducted in the absence of any commercial or financial relationships that could be construed as a potential conflict of interest.

## Footnotes

1 The learning effectiveness of the animations was tested with the reaction time task in a pilot-study with adults (*N* = 78) using Amazon’s Mechanical Turk. We translated the exact instructional materials designed for Dutch children for the English speaking adult sample. This pilot test showed that the animations were effective for learning (accuracy on the reaction-time task) as compared to no instruction [*t*(76) = -2.644, *p* = 0.010, Cohen’s *d* = 0.602 (large effect)]. No effects were obtained for solving speed on the RT-task.

2 http://charlyeielts.nl/bodyanalogy/materials.html

3 https://osf.io/4pkjx/
